# Feasibility of single breath-hold left ventricular function with 3 Tesla TSENSE acquisition and 3D modeling analysis

**DOI:** 10.1186/1532-429X-10-24

**Published:** 2008-05-21

**Authors:** Alistair A Young, Brett R Cowan, Stefan O Schoenberg, Bernd J Wintersperger

**Affiliations:** 1 Auckland MRI Research Group, University of Auckland, Auckland, New Zealand; 2 Institute of Clinical Radiology and Nuclear Medicine, University Medical Center Mannheim, University of Heidelberg, Mannheim, Germany; 3 Department of Clinical Radiology, University Hospitals Munich-Grosshadern Campus, University of Munich, Munich, Germany

## Abstract

**Background:**

A single breath-hold evaluation of ventricular function would allow assessment in cases where scan time or patient tolerance is limited. Spatiotemporal acceleration techniques such as TSENSE decrease cardiovascular MR acquisition time, but standard slice summation analysis requires enough short axis slices to cover the left ventricle (LV). By reducing the number of short axis slices, incorporating long axis slices, and applying a 3D model based analysis, it may be possible to obtain accurate LV mass and volumes. We evaluated LV volume, mass and ejection fraction at 3.0T using a 3D modeling analysis in 9 patients with a history of myocardial infarction and one healthy volunteer. Acquisition consisted of a standard short axis SSFP stack and a 15 heart-beat single breath-hold six slice multi-planar (4 short and 2 long axis) TSENSE SSFP protocol with an acceleration factor of *R *= 4.

**Results:**

Differences (standard minus accelerated protocol mean ± s.d.) and coefficients of variation (s.d. of differences as a percentage of the average estimate) were 7.5 ± 9.6 mL and 6% for end-diastolic volume (p = 0.035), 0.4 ± 5.1 mL and 7% for end-systolic volume (p = NS), 7.1 ± 8.1 mL and 9% for stroke volume (p = 0.022), 2.2 ± 2.8% and 5% for ejection fraction (p = 0.035), and -7.1 ± 6.2 g and 4% for LV mass (p = 0.005), respectively. Intra- and inter-observer errors were similar for both protocols (p = NS for all measures).

**Conclusion:**

These results suggest that clinically useful estimates of LV function can be obtained in a TSENSE accelerated single breath-hold reduced slice acquisition at 3T using 3D modeling analysis techniques.

## Background

Although Cardiovascular Magnetic Resonance (CMR) imaging provides accurate assessment of left ventricular (LV) mass and volumes [1], the data acquisition is relatively lengthy compared with computed tomography or echocardiography. The standard protocol for CMR LV volume and mass calculation is steady-state free precession (SSFP) image acquisition in contiguous (or with a small inter-slice gap) short axis slices, each slice being acquired in a separate breath-hold, so as to cover the entire LV [2] from the apex through the base into the left atrium. The endocardial and epicardial contours of the LV are then semi-automatically defined in each slice at end-diastole and end-systole, followed by manual editing where required, and the LV volume and mass calculated by slice summation [3]. However, this protocol typically requires 10–15 minutes for image acquisition.

Evaluation of ventricular function from a single breath-hold acquisition would be advantageous in cases where scan time is at a premium, for example where patient tolerance is poor, or where ventricular function is not the primary clinical goal but a rapid estimate of ventricular function would add clinical value. Also, rapid evaluation of ventricular function is required where transient effects are being studied, for example under pharmacological or exercise stress conditions, or in transient alterations to pre- or after-load conditions. Furthermore, single breath-hold acquisitions avoid errors due to variability in diaphragmatic position, and therefore cardiac position, between successive breath-holds.

Fast imaging techniques have been proposed to reduce the acquisition time, enabling multi-slice acquisitions within a breath-hold by utilizing parallel [4] and real time SSFP imaging [5]. Temporal acceleration approaches have also been proposed, such as UNFOLD [6] and TSENSE [7] which offer further improvement. However, these methods typically suffer from loss of SNR, as well as spatial and temporal resolution. Wintersperger *et al. *[8] showed that acquisition at 3.0T compensated for loss in SNR with TSENSE acceleration. However, it was not possible to acquire sufficient short axis slices within a single breath-hold at acceptable image resolution. Single breath-hold acquisitions comprising a reduced number of slices, without compromising image resolution, may be possible using a 3D modeling analysis procedure.

This study sought to evaluate left ventricular volumetric parameters 3.0T, using 3D guide-point modeling analysis [9] and single breath-hold multiplanar TSENSE acquisition. It was hypothesized that analysis of single breath-hold TSENSE studies with reduced slices using 3D modelling would provide accurate and reproducible functional information compared with a reference non-accelerated acquisition of contiguous short axis slices in separate breath-holds, at 3.0T.

## Methods

Ten individuals (eight male, aged 28–63 years, mean 49), comprising nine patients and one healthy volunteer, were enrolled in the study. All patients had history of myocardial infarction with documented regional wall motion abnormality (mainly in the mid to apical antero-septal regions) and/or impaired systolic global LV function. Written informed consent was obtained from all subjects and the appropriate regulatory authorities approved the study protocol. Comparisons of accelerated and non-accelerated acquisitions using slice summation analyses in this group have been reported previously [8].

**Figure 1:**
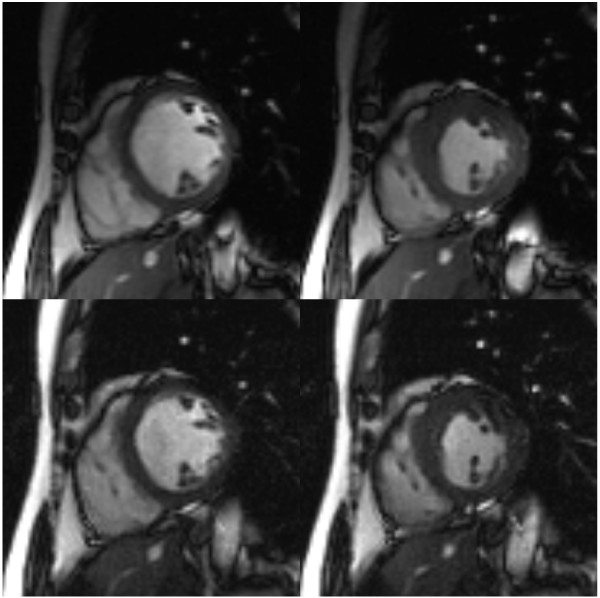
Midventricular short axis images from the standard acquisition (upper) and TSENSE accelerated single breath-hold acquisition (lower), at end-diastole (left) and end-systole (right), from the same patient.

Subjects were imaged on a 3.0T whole body scanner (Tim Trio, Siemens Medical Solutions, Erlangen). A segmented SSFP scout sequence simulating varying frequency offsets (-200 to 200 Hz) to the adjusted resonance frequency was used along a cardiac four chamber view and the shift with least image artifacts was implemented for all subsequent CINE SSFP data acquisitions [10]. Standard non-accelerated SSFP cine acquisitions were performed to obtain full coverage with one slice per breath-hold (one horizontal long axis and ~10 short axis slices). An accelerated SSFP acquisition using TSENSE with an acceleration factor of *R *= 4 was then performed, obtaining six slices (horizontal and vertical long axis slices plus four short axis slices spaced over the LV) within a single breath-hold. The short axis slices were planned according to the AHA segmental model – basal, midventricular and apical, with an additional slice through the apex [11]. As far as possible, imaging parameters were set to the same values for both protocols to facilitate comparison: typical parameters were TR/TE/FOV 3.0 ms/1.5 ms/360 mm, image matrix 192 × 117, spatial resolution 1.9 × 2.5 mm, temporal resolution 52 ms, slice thickness 8 mm. The flip angle was maximized with individual adaptation to normal operating mode specific absorption rate (SAR) limits of 2 W/kg with a maximum of 60° (range 39–51° for the standard acquisition, 55–60° for the accelerated acquisition). Acquiring 17 lines/segment, breath hold duration was 8 heart-beats for the single slice acquisitions (total duration 7–14 minutes) and 15 heart-beats for the accelerated multi-slice acquisition. The number of cardiac phases acquired ranged from 13–22 depending on the subject's breath-hold tolerance and heart rate. Representative images are shown in Figure 1.

All 20 studies were assigned a random number and then analyzed by a single observer (four years experience) in random order blinded to patient identifier, using guide-point modeling (CIM version 4.6, Auckland MRI Research Group, University of Auckland, New Zealand). The analyst could not be blinded to the acquisition method, due to the different number of slices in each method. Based on the study analyses, left ventricular end-diastolic volume (EDV), end-systolic volume (ESV), stroke volume (SV), ejection fraction (EF) and myocardial mass were calculated by numerical integration of the model surfaces. LV mass was calculated for every phase and then averaged (assuming a myocardial density of 1.05 g/ml). In order to determine intra-observer reproducibility, all studies were randomized a second time and re-analyzed by the same observer, blinded to previous results. Accuracy (relative to the non-accelerated acquisition) and reproducibility (intra-observer errors) were quantified for LV mass, EDV, ESV, SV and EF. A second blinded observer also analyzed all studies in order to estimate the inter-observer variability.

### Statistics

Repeated measures ANOVA was performed using both analyses of both acquisitions. Fixed effects in the ANOVA model were repeated analyses, acquisition protocol (i.e. "standard" acquisition versus "accelerated" single breath-hold acquisition), and their interaction. A significant interaction between repeated analysis and acquisition protocol would indicate that the reproducibility of the estimate was dependent on the acquisition method. If significant effects were found, Scheffé post-hoc tests were performed to test for significant differences between each of the acquisition protocols and repeated analyses. Significance was defined as p < 0.05.

## Results

**Figure 2:**
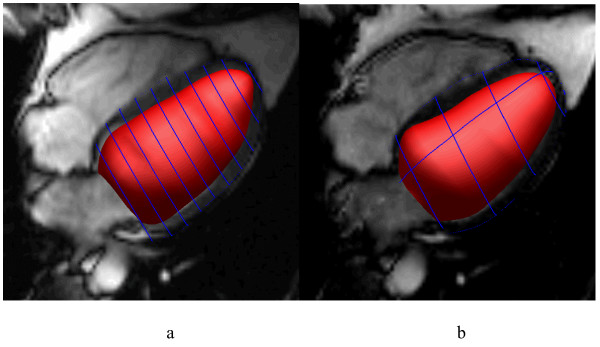
Mathematical representation of the LV derived from guide-point modeling at end-diastole, shown with an intersecting four chamber long axis slice, for a) standard protocol and b) reduced slice TSENSE accelerated single breath-hold acquisition. The endocardial surface is shaded red and intersections of the epicardial surface with the image slices are shown as blue lines.

**Figure 3:**
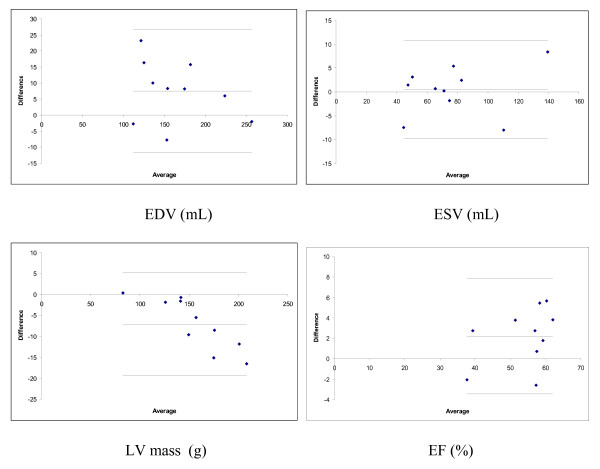
Bland Altman plots for EDV, ESV, LV mass and EF. Plots show difference (standard minus accelerated protocol) vs average (of both protocols). Lines show mean difference and 2 sd of differences.

Figure 2 shows typical results of the guide point modeling process in the standard and accelerated protocols (see Additional file 1 for a movie of the latter). Table 1 shows the mass and volume results for each acquisition protocol. Bland-Altman plots are shown in Figure 3. Table 2 shows the intra-observer errors obtained for each acquisition protocol.

**Table 1:** Functional parameters (mean ± s.d.) for each acquisition protocol (average of repeat analyses) and difference (standard minus accelerated protocol).

	**EDV**	**ESV**	**SV**	**EF**	**LV mass**
	(ml)	(ml)	(ml)	(%)	(g)
**Standard**	168 ± 34	77 ± 30	91 ± 26	55 ± 9	152 ± 34
**Accelerated**	160 ± 48*	76 ± 30	84 ± 27*	53 ± 8*	159 ± 40*
**Difference**	7.5 ± 9.6	0.4 ± 5.1	7.1 ± 8.1	2.2 ± 2.8	-7.1 ± 6.2

EDV = end-diastolic volume, ESV = end-systolic volume, SV = stroke volume, EF = ejection fraction, LV mass = left ventricular mass. * p < 0.05 versus the standard protocol by ANOVA.

**Table 2:** Intra-observer error for each acquisition protocol (mean ± s.d.).

	**EDV**	**ESV**	**SV**	**EF**	**LV mass**
	(ml)	(ml)	(ml)	(%)	(g)
**Standard**	-2.4 ± 4.4	-2.3 ± 4.5	-0.1 ± 5.9	0.9 ± 2.2	6.9 ± 9.4
**Accelerated**	-1.2 ± 3.5	-1.1 ± 3.9	-0.1 ± 6.4	0.5 ± 3.6	4.9 ± 8.7

For EDV, the only significant effect on ANOVA was acquisition protocol (EDV was underestimated by 5% in the accelerated protocol, p = 0.035). On post-hoc tests this difference was significant in both analyses (Scheffé p < 0.01 for both). For ESV, no overall effects were significant on ANOVA. For SV, the only significant effect was acquisition protocol (8% underestimation, p = 0.022). On post-hoc tests this difference was significant in both analyses (Scheffé p < 0.05 for both). For EF, the only significant overall effect was acquisition protocol (4% underestimation, p = 0.035). However, on post-hoc tests this difference was not significant in each analysis (Scheffé p = NS for both). For LVM, significant effects were found due to repeated analysis (4% difference, p = 0.015) and acquisition protocol (5% overestimation, p = 0.005). However, on post-hoc tests, no differences were significant between analyses in each acquisition protocol, or acquisition protocols in each analysis (all Scheffé p = NS).

For all functional parameters, the interaction between repeated analysis and acquisition protocol was not significant, indicating that intra-observer error was similar in both the standard and accelerated protocols.

Inter-observer errors are shown in Table 3. There were no significant differences between observers.

**Table 3:** Inter-observer error for each acquisition protocol (mean ± s.d.).

	**EDV**	**ESV**	**SV**	**EF**	**LV mass**
	(ml)	(ml)	(ml)	(%)	(g)
**Standard**	-1.0 ± 7.2	-1.0 ± 5.5	0.0 ± 6.7	0.4 ± 3.2	3.9 ± 8.2
**Accelerated**	3.3 ± 5.0	2.3 ± 4.8	1.0 ± 5.8	-0.7 ± 3.5	3.8 ± 9.0

## Discussion

Although current acquisition protocols enable accurate estimation of LV function, the time requirements of these protocols are relatively large. Due to practical constraints such as patient tolerance, available scan time and patient comfort, more rapid evaluation of ventricular function is desirable. Rapid acquisition of ventricular function would also facilitate investigation of transient phenomena including pharmacological or exercise stress studies, and preload and afterload manipulations, for example during performance of the Valsalva maneuver. In order to achieve faster image acquisition, compromises may need to be made in the areas of LV coverage, spatial and temporal resolution, and SNR. In the present study, the first compromise was ameliorated by 3D modeling, the second by TSENSE and the third by higher field strength (3T).

Single breath-hold acquisition for LV function has been previously evaluated using a real-time SSFP protocol [5]. In order to enable ventricular coverage, spatial resolution was reduced to ~4.2 mm/pixel in the phase encode direction, and temporal resolution was ~91 ms, allowing 9 short axis slices to be acquired in a 20–30 second breath-hold. The mean differences ( ± s.d. of the differences) with respect to a standard SSFP protocol in 12 volunteers and 8 patients were 4 ± 9 mL for EDV, 4 ± 15 mL for ESV, 0 ± 14 mL for SV, 1.6 ± 7% for EF and 0 ± 8 g in LV mass. These results correspond to higher coefficients of variation than found in the present study (except for EDV), but reduced bias in EDV, SV, EF and LV mass. Another study [10] using a single breath-hold real time SSFP protocol with spatial resolution ~3.5 mm, temporal resolution ~77 ms, acquiring 7 slices in 14 heart-beats, found 3 ± 6 mL for EDV, -4 ± 5 mL for ESV, 6 ± 4 mL for SV, 4 ± 3% for EF in 25 patients (LV mass not reported). These results correspond to lower coefficients of variation but higher bias than the present study.

Parallel imaging methods were used in [12] to improve the temporal resolution of real time single breath-hold SSFP techniques. A 10 short axis slice acquisition in 18–22 heart-beats was performed with spatial resolution of ~4.2 mm and temporal resolution of ~50 ms with *R *= 2. Comparison with a standard SSFP protocol showed differences of 4 ± 12 mL for EDV, 1 ± 5 mL for ESV, 3 ± 10 mL for SV, 0 ± 2% for EF in 18 patients. These results are comparable with the present study; however the present protocol had a shorter breath-hold duration and higher spatial resolution.

Parallel imaging has also been exploited in 3D SSFP techniques to achieve single breath-hold acquisition of LV function. In [13], SENSE 3D SSFP with *R *= 3 allowed a 14 short axis slice 3D acquisition (with 2 slices discarded) in 18–20 beats, ~2.5 mm/pixel spatial resolution and ~50 ms reconstructed temporal resolution. The mean differences were 3 ± 15 mL in EDV, 1 ± 11 mL in ESV, 1 ± 4% in EF, and 1 ± 11 g in LV mass in 15 patients and 22 volunteers. These results correspond to higher coefficients of variation than the present study.

Single breath-hold 4D kt-BLAST accelerated techniques have been recently investigated for ventricular function [14,15]. In [14], a 12–14 short axis slice 3D acquisition, with 2.4 mm spatial resolution and 34 msec temporal resolution, was performed in 16–17 seconds, with an additional 8 seconds required for training data. In 40 patients, mean differences were approximately 4.9 ± 8 mL in EDV and 1.8 ± 6 mL in ESV. In [15], 14–16 short axis slices were acquired, with ~3 mm spatial resolution and 38 msec temporal resolution in 15 seconds, with an additional 10 second breath-hold required for training data. In 17 healthy volunteers, mean differences were approximately 5 ± 5 mL in EDV and 1 ± 6 mL in ESV, 0.5 ± 3% in EF, and -1 ± 4 g in LV mass. In comparison with the current reduced slice protocol, 3D kt-BLAST methods resulted in similar accuracy and improved ventricular coverage, with slightly longer breath-hold duration and reduced in-plane resolution, and the requirement for a training dataset acquisition. Short axis oriented 3D acquisitions with 6–8 mm effective slice thickness can also result in less accurate delineation of the basal and apical areas, due to partial voluming, compared with long axis images.

Fieno *et al. *[16] applied a geometric interpolation of long and short axis information to enable a reduction in the number of slices acquired in a single breath-hold. A reduced slice SSFP acquisition was performed in a 20 heart-beat breath-hold by reducing the spatial and temporal resolution of a standard segmented protocol to ~2.7 mm/pixel, and 60 ms respectively. Five slices were acquired, three short axis and two long axis, in 62 patients. LV volume was determined using an ellipsoidal interpolation of image contours. The mean differences were 9 ± 15 mL in EDV, 6 ± 12 mL in ESV, and 2 ± 5% in EF (SV and LV mass not reported). These results correspond to higher bias and coefficients of variation than the present study.

Guide point modeling has been proposed as an accurate and efficient tool for LV volumetric analysis [9]. The method creates a mathematical representation of the LV by optimizing a generic 3D beating finite element model to the specific patient images, thereby maintaining spatially and temporally consistent LV geometry and motion [9]. LV volumes are calculated by numerical integration of the endo- and epicardial curved surfaces represented by the finite element model, and are therefore less dependent on the positioning of the images. The motion of the base is accurately calculated by tracking the mitral valve plane position in one or more of the long axis images. Incorporation of long axis slices enables better definition of the apical and basal regions, which are often poorly seen in short axis images due to partial voluming and through plane motion [17]. This method has been previously validated against slice summation analysis methods in patients with cardiac disease, against post mortem results in animal studies, and against phase contrast velocity estimates of stroke volume (SV) [9].

The results of the present study showed that EDV was underestimated by 5% in the accelerated acquisitions, leading to underestimations in SV (8%) and EF (4%). Interestingly, the estimate of ESV was not significantly different between accelerated and standard acquisitions. This is in accordance with a previous investigation in the same subjects [8] using a two breath-hold, 10 short axis slice TSENSE accelerated acquisition protocol incorporating a slice summation analysis. Similar to the present study, the double breath-hold slice summation protocol also showed an underestimation of EDV but not ESV. These results suggest that the underestimation of EDV is due to the accelerated acquisition and not the reduction in the number of slices acquired. Since only EDV is affected, it may be hypothesized that the loss in SNR with accelerated acquisition leads to inferior delineation of small subendocardial trabeculations. Another possible cause may be the temporal filtering incorporated in the TSENSE method [7].

LV mass was overestimated by 5% within the accelerated protocol, and reproducibility was worse for LV mass than all other volumetric parameters. One possible cause for the increased variability in LV mass may be decreased myocardial signal due to reduced flip angle to comply with SAR constraints, leading to increased uncertainty in the location of the epicardial contour relative to the low signal in the lungs. The Bland Altman plot for LV mass shows a tendency for increased difference with larger mass, possibly due to the reduced SNR in the TSENSE acquisition, as the maximum error of 8% may be attributed to a displacement of the epicardial contour of less than half a pixel.

The application of the reduced slice protocol is intended only for cases in which a full slice protocol is not possible or desired. The reduced slice protocol is not appropriate for applications such as research trials with cardiac mass and volume endpoints in which inter-study variability is very important. The reduced slice protocol is unlikely, on its own, to be as effective as a full stack of short axis cines for the assessment of regional wall motion abnormalities or, when supplemented by late gadolinium enhancement imaging, for viability assessment. A reduced slice accelerated acquisition protocol could also be applied to the right ventricle, and geometric models of right ventricular shape and motion are being constructed. However, the complex geometry of the right ventricle, with separated inflow and outflow tracts, may result in decreased accuracy using a reduced slice protocol. In this case it might be advantageous to trade some of the short axis slices for more long axis slices, for example in the inflow and outflow tracts.

A limitation of the current study was the small number of patients scanned. This study was performed mainly in patients with a history of myocardial infarction, since it was expected that regional abnormalities in LV shape and motion typically observed in such patients could lead to decreased accuracy when assessed with a reduced slice acquisition protocol. Also, the myocardial signal intensity may have been compromised by the need to comply with SAR constraints and accelerate the acquisition. Future improvements in SNR with higher acceleration factors may be achieved with a higher density of receive coils [18], which may also enable 2D acceleration factors [19].

## Conclusion

Single breath-hold ventricular function evaluation involves a trade-off in spatial resolution, temporal resolution, and SNR. In this study TSENSE accelerated multi-planar segmented cine acquisition enabled high spatial and temporal resolution while reduced ventricular coverage was compensated by 3D modeling, and SNR improved by higher field strength. These preliminary data demonstrate the feasibility of single breath-hold LV assessment at high spatial and temporal resolution with acceptable reproducibility at 3.0T.

## Supplementary Material

Additional file 1 Six slice TSENSE single breath-hold acquisition with LV model. A typical result of the beating LV model customized to six slices acquired in a single breath-hold TSENSE acquisition. The four chamber long axis SSFP image is shown with the LV model endocardial surface shaded in red. The blue lines are the intersections of the LV epicardial surface with the image planes. Note apical and basal motions are well characterized by the long axis slices. Click here for file
